# Structural characterization of a hypothetical protein: a potential agent involved in trimethylamine metabolism in *Catenulispora acidiphila*

**DOI:** 10.1007/s10969-014-9176-z

**Published:** 2014-02-22

**Authors:** Ekaterina V. Filippova, Chi-Hao Luan, Sara F. Dunne, Olga Kiryukhina, George Minasov, Ludmilla Shuvalova, Wayne F. Anderson

**Affiliations:** 1 Department of Molecular Pharmacology and Biological Chemistry, Northwestern Feinberg School of Medicine, Chicago, IL 60611 USA; 2 High Throughput Analysis Laboratory, Department of Molecular Biosciences, Northwestern University, Evanston, IL 60208 USA

**Keywords:** Trimethylamine, NTF2-like superfamily, Caci_0382, X-ray crystal structure, Structural genomics

## Abstract

*Catenulispora acidiphila* is a newly identified lineage of actinomycetes that produces antimicrobial activities and represents a promising source of novel antibiotics and secondary metabolites. Among the discovered protein coding genes, 68 % were assigned a putative function, while the remaining 32 % are genes encoding “hypothetical” proteins. Caci_0382 is one of the “hypothetical” proteins that has very few homologs. Sequence analysis shows that the protein belongs to the NTF2-like protein family. The structure of Caci_0382 demonstrates that it shares the same fold and has a similar active site as limonene-1,2-epoxide hydrolase, which suggests that it may have a related function. Using a fluorescence thermal shift assay, we identified stabilizing compounds that suggest potential natural ligands of Caci_0382. Using this information, we determined the crystal structure in complex with trimethylamine to provide a better understanding of the function of this uncharacterized protein.

**Electronic supplementary material:**

The online version of this article (doi:10.1007/s10969-014-9176-z) contains supplementary material, which is available to authorized users.

## Introduction

Defining the function of gene families that lack significant sequence homology to previously characterized genes is one of the goals of the Midwest Center for Structural Genomics (MCSG). As a part of the MCSG project, genome sequence information was used to select target proteins from a phylogenetically diverse set of genomes, including species that may affect global carbon cycling, species that play a role in the degradation of lignocellulosic material and those with a rich metabolic potential. *Catenulispora acidiphila* is a small, free living, non motile, gram positive acidophilic aerobic bacterium from soil for which the genome sequence was completed a few years ago [1]. It is of interest to MCSG because this organism plays an important role in the carbon cycle, can produce secondary metabolites and may be a novel antibiotic producer [2].

*Catenulispora acidiphila DSM 44928* contains 9056 predicted protein-coding genes. *Caci_0382* is one of these genes, which encodes for a 134 amino-acid putative protein with a molecular weight of 14.3 kDa. While the exact function of Caci_0382 is not known, sequence comparison suggests that the protein belongs to the nuclear transport factor 2 (NTF2) superfamily. This family contains thousands of functionally divergent single-domain and multi-domain proteins, including NTF2 members that mediate the nuclear import of Ran-GDP [3]; Ca^2+^/calmodulin-dependent protein kinases II (CaMKII) that are involved in many signaling cascades [4]; Δ^5^-3-ketosteroid isomerases (KSI) that catalyze the isomerization of Δ^5^-3-ketosteroid to Δ^4^-3-ketosteroid [5]; limonene-1,2-epoxide hydrolases (LEH) which participate in limonene and pinene degradation [6]; SnoaL-like polyketide cyclase (SnoaL) involved in nogalamycin biosynthesis [7]; and others. Proteins of known structure in the NTF2-like family form a cone-like fold of three α-helices and a six-stranded β-sheet that contains a deep cavity for the substrate-binding pocket. Typically, the active site of NTF2-like proteins accommodates primarily hydrophobic ligands.

The three-dimensional crystal structure of Caci_0382 from *Catenulispora acidiphila*, described here, reveals that the protein forms a homodimer, wherein each subunit contains the NTF2 conserved structural fold with a similar binding site to LEH. Using a fluorescence thermal shift (FTS) assay, we identified compounds from a chemical library that point to the potential biological ligands of Caci_0382. We determined a crystal structure of Caci_0382 with one of those compounds—trimethylamine (TMA). The details of the protein architecture and the TMA binding site are presented. Bioinformatics, structural analysis, ligands discovered by FTS analysis and the resulting insights into the possible biological function of Caci_0382 are discussed.

## Materials and methods

### Sequence and structural analysis

PSI-BLAST [8] was used for a sequence-based search against the non-redundant NCBI database. Multiple sequence alignments were constructed with MUSCLE [9] for the sequence homologs of Caci_0382 from *Catenulispora acidiphila* *DSM 44928* that were identified by the database search. The most related sequences were aligned using CLUSTALW [10] and formatted using ESPript [11]. The DALI [12] and ProFunc [13] web servers were used to identify three-dimensional crystal structures of proteins that share 3-D structural similarity with Caci_0382.

### Protein cloning, expression and purification

The recombinant Caci_0382 protein from *Catenulispora acidiphila* *DSM 44928* was subcloned in the pMCSG57 vector that adds a 6 His-Tag at the N-terminus and was developed at MCSG [14]. Caci_0382 was expressed in *Escherichia coli* BL21-magic cells by isopropyl β-d-1-thiogalactopyranoside (IPTG) induction in High Yield M9 SeMet media kit (Medicilon Inc.) for selenomethionine-labeled Caci_0382 protein. The protein was purified by Ni affinity chromatography and further by size exclusion chromatography followed by gel filtration on Superdex 200 column (GE Healthcare, USA). The purified protein solution was concentrated in a buffer containing 10 mM Tris–HCl pH 8.3, 500 mM NaCl and 5 mM β-mercaptoethanol.

### Fluorescence thermal shift analysis

A robotic pipeline in the High Throughput Analysis Laboratory (HTAL) was used for protein ligand screening by fluorescence thermal shift (FTS) analysis. The pipeline used a Mosquito robot (TTP Labtech) for protein dispensing and a Biomek FX MP96 microliter robot to add screen conditions. Thermal scanning coupled with fluorescence detection was performed on a real-time PCR machine CFX384 (Bio-Rad Laboratories). The assay was run in 384-well PCR plates, using 2 μg protein per well in a 10 μl assay with Hepes buffer (20 mM Hepes, pH 7.5, 150 mM NaCl). The assay concentration for protein was 14 μM and that for Sypro Orange (Invitrogen) was 5X. One μl protein premixed with Sypro Orange was dispensed to a plate first and 9 μl screen condition added. Then the plate was sealed with optical seal, shaken, and centrifuged. The thermal scan was from 10 to 95 °C with a temperature ramp rate of 1.5 °C/min. The fluorescence was recorded every 10 s. Data analysis and report generation were performed by using the in-house software excelFTS of the HTAL.

The in-house libraries of 260 unique conditions were screened (Supplementary Table S1). Then a dose response test was performed on the hit compounds. The most prominent hit was TMA, for which the T_m_ shift was 16 °C at 100 mM. Subsequent dose-reponse screens found that the T_m_ was shifted by 3 °C by 0.1 mM TMA, clearly indicating specific binding.

### Crystallization

The sitting drop vapor-diffusion method was used for crystallization of purified Caci_0382 at 19 °C using Corning 96-well sitting-drop plates. Crystallization drops contained 1 μl of reservoir and 1 μl of protein solution (concentrated to 8.15 mg/ml). For co-crystallization Caci_0382 was incubated with 5 mM TMA for 30 min before screening of crystallization conditions. Screening kits Classics II, Classics Lite, PEG, PACT and JCSG + (QIAGEN Sciences, MD, USA) were used for crystallization trials. A well diffracting crystal of the apo-form was obtained in a solution containing 0.05 M Cadmium Sulfate, 0.1 M HEPES pH 7.5 and 0.5 M Na Acetate. Crystals of the complex with TMA were observed in a condition containing 0.1 M SPG buffer (the mixture of succinic acid, sodium dihydrogen phosphate, and glycine in the molar ratios 2:7:7) pH 5.0 and 25 % (w/v) PEG 1500. For data collection Caci_0382 crystals were flash cooled in liquid nitrogen using 25 % of sucrose as a cryo-protectant.

### Data collection and model building

Low temperature (100°K) X-ray diffraction datasets were collected from single crystals of the hypothetical protein Caci_0382 at the LS-CAT 21ID-G beamline at the Advanced Photon Source (Argonne, IL, USA). The data were indexed, integrated and scaled with the HKL-3000 program suite [15]. The high-resolution Caci_0382 apo-form structure was solved using the single-wavelength anomalous dispersion method (SAD). The structure of the complex with TMA was solved using the molecular replacement method with PHASER [16] and the apo-form structure as the model. Automatic model building was carried out with HKL-3000 [15]. The programs COOT [17] and REFMAC [18] were used for manual building and least-squares refinement of both structural models, respectively. Structural figures were produced using CCP4MG [19] and PyMOL [20]. Data collection, structure solution and refinement statistics are summarized in Table 1. Deposited structures were assigned following PDB codes: 4H3U (apo-form) and 4HVN (complex with TMA).

**Table 1:** Crystallographic parameters, X-ray data-collection and processing statistics

	Apo-form	Complex with TMA
Crystal parameters		
Space group	P6_1_	P2_1_
Cell dimensions:		
a, b, c (Å)	61.6, 61.6, 144.6	45.1, 61.1, 52
α, β, γ, (°)	90, 90, 120	90, 103.7, 90
Matthews coefficient (Å^3^/Da)	2.3	2
Solvent content (%)	46.4	38.9
Data collection^a^		
Wavelength (Å)	0.97856	0.97856
Resolution (Å)	50.0–1.15 (1.17–1.15)	30.0–1.95 (1.98–1.95)
R_merge_ (%)	5.4 (50.5)	8.5 (48.0)
No. of unique reflections	212,580	20,052
Mean redundancy	5.8 (5.6)	3.7 (3.4)
Overall completeness (%)	97.4 (95.7)	99.8 (99.1)
Mean I/σ_I_	34.7 (2.8)	20.9 (2.6)
Refinement residuals		
R_free_ (%)	14.3 (19.5)	21.8 (31.8)
R_work_ (%)	12.8 (17.1)	17.7 (27.5)
Completeness (%)	99.9 (99.5)	99.5 (94.2)
Model quality		
RMSD bond lengths (Å)	0.014	0.014
RMSD bond angles (°)	1.7	1.7
MolProbity Ramachandran distribution:		
Most favored (%)	100	100
Allowed (%)	NA	NA
Disallowed (%)	NA	NA
Mean main chain B value (Å^2^)	10.8	35.8
Mean overall B value (Å^2^)	14.6	38.5
Mean solvent B value (Å^2^)	33.1	40.8
Model contents		
Protomers in ASU	2	2
No. of protein atoms	1,955	1,950
No. of cadmium/chloride/acetate ions	7/7/4	NA
No. of water molecules	417	81

^a^Data for the highest resolution shell are given in parentheses. RMSD and ASU stands for root-mean-square deviations and asymmetric unit cell, respectively

## Results and discussion

### Sequence comparison and homology search analysis

**Fig. 1:**
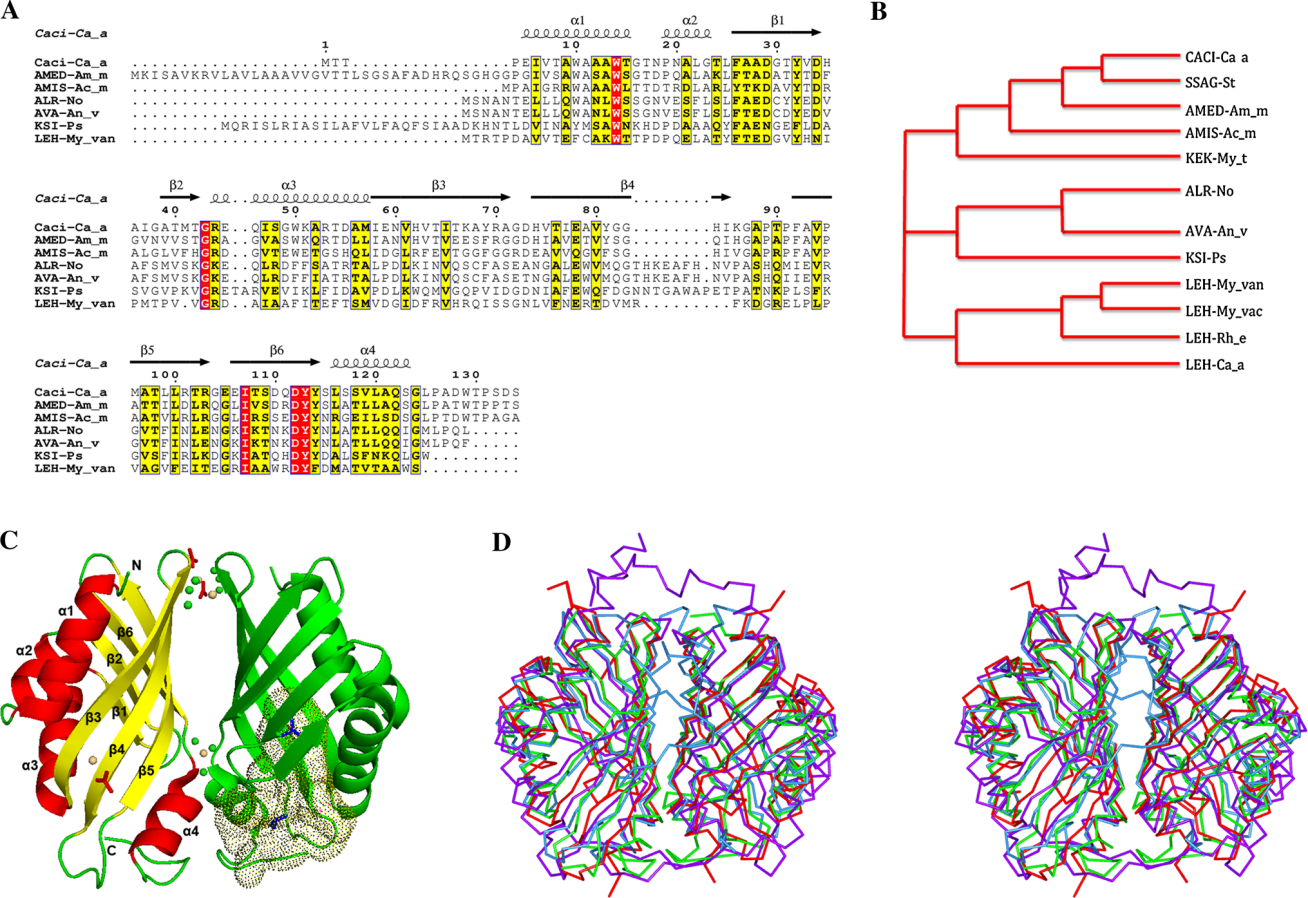
Sequence alignment and Overall structure of the Caci_0382 protein. **a** and **b** Sequence alignment and phylogenetic tree of Caci_0382 from *Catenulispora acidiphila DSM 44928* (Caci-Ca_a) and its homologs: SSAG_04049 from *Streptomyces sp. Mg1* (SSAG_St), AMED_3668 from *Amycolatopsis mediterranei U32* (AMED-Am_m), AMIS_27750 from *Actinoplanes missouriensis 431* (AMIS-Ac_m), ALR3729 from *Nostoc sp. PCC 7120* (ALR-No), Ava_1595 from *Anabaena variabilis ATCC 29413* (AVA-An_v), KSI-like protein from *Pseudomonas sp. GM78* (KSI-Ps), KEK_02356 from *Mycobacterium thermoresistibile ATCC 19527* (KEK-My_t), LEH from *Mycobacterium vanbaalenii PYR*-*1* (LEH-My_van), LEH from *Mycobacterium vaccae ATCC 25954* (LEH-My_vac), LEH from *Rhodococcus erythropolis* (LEH-Rh_e), LEH from *Catenulispora acidiphila DSM 44928* (LEH-Ca_a). Secondary structure elements of Caci_0382 are indicated above the sequence. Sequence homologies are highlighted by *red* background (identities) and *yellow* (similarities). **c** *Ribbon diagram* of the Caci_0382 dimer in complex with TMA (shown as the *stick blue* model in one subunit). Cadmium (*yellow*), chloride (*green*) and acetate (*red*) ions are shown as ball and stick model, respectively. The binding cavity in one subunit of the Caci_0382 structure is presented as *dots*. The secondary structure elements of the one subunit are labeled. **d** Stereo view of the structural homologs [LEH (*light blue*), NTF2 (*red*), and PHZB (*purple*)] superposed on the Caci_0382 dimeric structure (*green*)

Caci_0382 from *Catenulispora acidiphila DSM 44928* is annotated as a hypothetical protein of unknown function. A search for homologs of Caci_0382 was performed using PSI-BLAST [8] and the non-redundant NCBI database. This produced the list of only 11 sequences with significant alignments (E value >0.005). Only three homologs (SSAG-St, AMED-Am_me, AMIS-Ac_mi) share more than 40 % sequence identities, much higher than the rest of the proteins that had less than 23 % amino acid identities (Fig. 1a, b). All the close homologs that had high identities are hypothetical proteins from the *Actinobacteria* phylum. The list of remote homologs with identities less than 23 % also includes hypothetical proteins from *Cyanobacteria* species (AVA-An_v, ALR-No), KSI-like proteins from *Pseudomonas* species and several LEHs from *Mycobacteriaceae* species. It is interesting to note that all of the Caci_0382 homologs are distributed in species that could be found in soil.

The sequence comparison and a phylogenetic tree were generated based on the Caci_0382 sequence and its homologs (Fig. 1a, b). The sequence alignment excludes proteins with long sequences (>160 residues) and those with short overlaps (<60 residues). Caci_0382 homologous proteins are divided into three distinct but strongly correlated groups (Fig. 1b). Close homologs fell into the same group as Caci_0382. Hypothetical proteins related to KSI-like proteins comprise the second group. The third group is composed by LEHs. This close relationship between three groups could indicate that these proteins are likely to share similar functions. Interestingly, *Catenulispora acidiphila DSM 44928* genome analysis shows that there is a paralog to Caci_0382, Caci_0376 that encodes a LEH.

### Overall structure

The main features of the Caci_0382 structure are a six-stranded mixed β-sheet and four α helixes (Fig. 1c). Three α helices [α1 formed by residues 5–15, α2 by (19–23), α3 by (44–57)] are packed on one side of the structure and lie side by side with three long curved β strands [β3 (58–71), β4 (74–85), β5 (92–103)]. The fourth α helix is formed by residues 116–123 and acts as a structural extension of the three shorter strands [β1 (26–34), β2 (39–42), β6 (106–114)] on the opposite side of the Caci_0382 molecule. The overall structure resembles a cone with an inner cavity ~20 Å long.

There are two molecules per asymmetric unit and they appear to form a homodimer with the subunits related by a twofold axis in both crystal forms (Fig. 1c). Structural comparison reveals that the Caci_0382 structure in apo-form and in complex with TMA are very similar and could be aligned with an average RMSD value of 0.4 Å for individual subunits (131 Cα atoms). The interface between the subunits of the apparent dimer is the surface formed by strands β3–β6 and helix α4. The total area of the contact surface is 809 Å^2^ and it has predicted favorable interaction energy with a ΔG of −9.5 kcal/mol. Overall, there are 24 interface residues that form 7 hydrogen bonds and 91 non-bonded contacts. The dimer suggested by the crystal structure of Caci_0382 is in a good agreement with gel-filtration chromatography (data not shown). It is not clear, however, if dimerization is important for the function of this protein.

In the apo-form structure, well-ordered cadmium ions were identified. Two cadmium ions are bound to the surface residues H61 and H74, and one to oxygen atoms of the main chain of residue Y114 between the protein subunits (Fig. 1c). The metal ions have octahedral coordination and other ligands include chloride and acetate ions.

### Comparison with known PDB structures and functions

A search of homologous structures by the Secondary Structure Matching (SSM) program DALI [12] revealed several structures with similar fold to Caci_0382 (Table 2). The highest ranked protein based on DALI Z-score is an LEH [6]. There are a number of related structures with Z score above 15 as listed in Table 2. However, none of these proteins display significant amino acid sequence conservation (all are less than 20 % sequence identity). All homologous structures are dimers with similar interactions, except for the dimeric structure of SnoaL. The interaction interface between SnoaL subunits is small (590 Å) with only three hydrogen bonds [7]. However, compared to the other protein structures, the SnoaL dimer has more complete burial of the active sites. This might mean that substrate binding (or product release) is coupled to significant conformational changes in the SnoaL dimer interface [7].

**Table 2:** Structural homologs of Caci_0382

Protein name	Z score	RMSD (Å)	PDB code	Number of aligned residues/number of residues	Identity (%)
Limonene-1,2-epoxide hydrolase	16.5	2.2	1NWW	128/145	17
Polyketide cyclase	15.9	1.7	3I0Y	119/135	20
NTF2-like protein	15.7	2.0	3EC9	122/130	19
Phenazine biosynthesis protein	15.6	2.1	3FF0	124/154	13
Phenazine biosynthesis protein A/B	15.5	2.1	3EX9	124/151	14
Polyketide cyclase	15.4	2.0	3F7X	119/133	17
Δ^5^-3-ketosteroid isomerase	15.4	2.3	3T8 N	117/130	17

The variations at N- and C-termini and the loops surrounding the ligand-binding cavity between the superposed proteins have been observed (Fig. 1d). In all structures the loop between strands β5–β6 exhibits conformational differences. Compared to the Caci_0382 structure, phenazine biosynthesis protein (PHZB) has conformational changes in the loop between strands β4–β5. For NTF2, changes have also been found in the loop between β1 and β2. These two loops in the structures are close to the binding cavity of the protein and the structural differences are likely to be related to their specific ligands. Compared to the other protein structures, PHZB has a long C-terminus that comes across the binding site from the opposite subunit. This extension acts as a “flexible lid” and is critical for activity and stability of PHZB [21]. The LEH structure shows the smallest structural differences compared to Caci_0382. The N-terminus of LEH is longer and runs close to the dimer interface changing the area of the contact interactions between protein subunits (Fig. 1d).

Looking for more proteins that might have a similar function, an alternative reverse template search using the ProFunc server [13] was performed. With a high similarity score (321.0) the program identified the structure of LEH (PDBID 1NWW) as having a conserved group of residues (F47, Y53, D132) that closely match residues (F26, Y32, D112) in the Caci_0382 structure. Residues Y53 and D132 are highly conserved in LEH enzymes and are implicated in catalysis [6]. Therefore, this search suggests that Caci_0382 could be a distant homolog to LEH and the residues involved in the functional site have been well conserved over evolution despite a divergence in the sequence.

### Identification of potential biological ligands by FTS

**Fig. 2:**
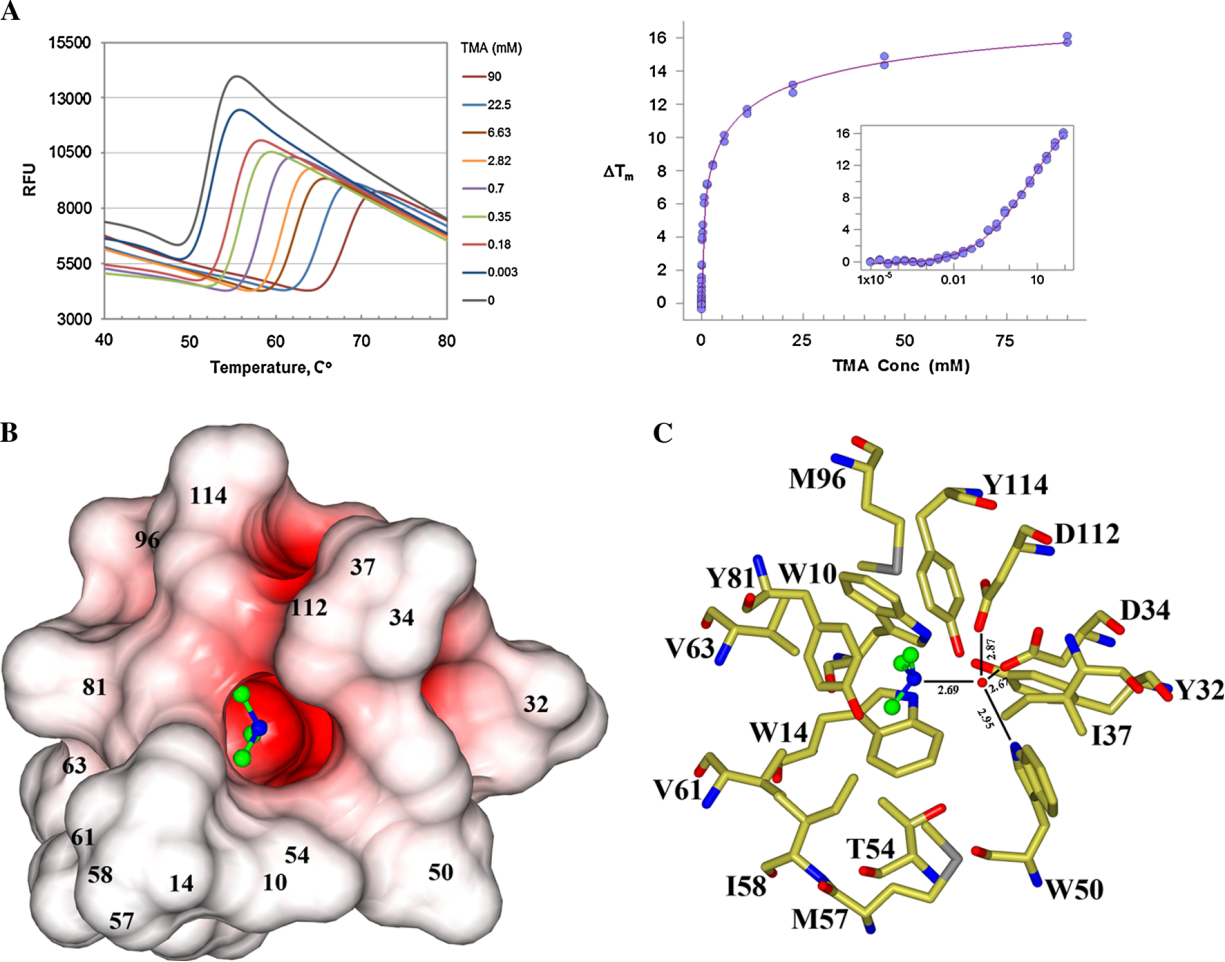
Fluorescent thermal shift analysis and TMA binding site. **a** Melting curves of Caci_0382 versus TMA concentration (*left*) and plots of ΔT_m_ versus TMA concentration (*right*). The TMA concentration of the *inset* is in log scale. The protein has a very steep unfolding transition. Specific binding of TMA is indicated by the significant transition temperature shift, which is 3 °C at 0.1 mM TMA. After an initial sharp rise, increasing TMA concentration shifts the transition progressively at a slower rate. However the plateau was never reached at the concentrations tested, suggesting multi-site binding of TMA with different affinities. **b** Electrostatic surface of the TMA (ball-and-stick model) binding pocket. The surface was created by program CCP4MG [19] and colored by surface potential charge scaled from negative in *red* (−0.5 V) to positive in *blue* (+0.5 V); **c** position of the TMA in the active site. Carbon and nitrogen atoms of the TMA colored in *green* and *blue*, respectively. In the model oxygen atoms of the surrounding residues are colored in *red*, nitrogen in *blue*, carbon in *gold* and sulfur in *grey*. H-bonds are shown as *lines*

The FTS assay was used to discover small molecule ligands for Caci_0382. By screening a 260 molecule in-house library, a number of molecules were found to cause a positive T_m_ shift to Caci_0382. The most prominent hit was TMA, for which the T_m_ shift reaches 16 °C at 100 mM. Melting curves of Caci_0382 in a subsequent dose–response experiment with TMA clearly demonstrated specific binding, as shown in Fig. 2a, b. TMA HCL and Imidazole (T_m_ = 16.67 and T_m_ = 10.26) were selected as potential ligands of Caci_0382. The crystallization trials using these compounds with Caci_0382 led to the identification of conditions that yielded diffraction quality crystals of Caci_0382 protein with TMA.

### Characterization of the TMA-binding pocket

A large cone-like cavity with an open entrance and a deep, narrow channel, going into center of the β-barrel is present in the Caci_0382 structure (Fig. 1c). The inner surface of the cavity is lined by residues: W10, W14, Y32, D34, I37, W50, R53, T54, M57, I58, V61, V63, Y81, I85, M96, D112, Y114, L116, V119, L120, W129, T130, P131. Completely buried tyrosine and aspartate residues of this channel create a negative electrostatic potential on the inner surface of this pocket (Fig. 2b). The negative potential and the size of the channel suggest that Caci_0382 may prefer to bind small positively charged ligand(s) such as the TMA and Imidazole identified in the FTS experiments. Two TMA molecules are bound in this predominantly hydrophobic pocket in the structure of Caci_0382 in complex with TMA. One molecule (TMA1) is located at the entrance of the pocket and the second (TMA2) binds at the bottom of the cavity, similar to what is observed in structures of proteins from the NTF2-like family with known ligands bound [5–7]. All interactions of the TMA2 with Caci_0382 are hydrophobic and van der Waals. It is interesting to note that the TMA2 binding site has approximate twofold non-crystallographic symmetry. Six related residues in the TMA2 binding pocket could be identified: W10–W50, Y32–Y81, and M57–M96. TMA2 sits in the middle of the pocket on this approximate twofold axis. The three CH3 groups of the ligand point to hydrophobic protein side chains: V61, V63, M96, and to the side chain of Y81 (Fig. 2c). The nitrogen NE1 atom of TMA2 makes hydrogen bonds through a water molecule with the OD2 atoms of residues D34 and D112, and with the NE1 atom of W50.

Sequence and structural comparisons with known structures from the NTF2-like protein family suggests that Caci_0382 is probably related in function to LEH as some of the catalytic residues (Y32, D112) appear to be conserved. The position of the water molecule that makes hydrogen bonds to D34, D112 and W50 in the Caci_0382 binding site could be a hydrolytic water and D34 a general acid catalyst as both are conserved in the LEH structure and play an important role in catalysis [6]. However, most of the residues (N55, R99, D101, W130) implicated in catalysis in LEH bear no similarity to Caci_0382. There are also differences in hydrophobic residues that line the walls of the ligand cavity and this may reflect differing characteristics of the natural substrates.

## Conclusions

In conclusion, through sequence analysis we have learned that Caci_0382 is only found in a small number of bacteria that could be identified from soil. The protein structure characterization suggests that the Caci_0382 structure is similar to LEH and that it shares a similar binding cavity with a conserved group of residues implicated in LEH catalytic activity. We discovered using a FTS assay that several positively charge molecules like TMA stabilize the Caci_0382 protein. The Caci_0382 complex structure with TMA sheds light on a likely function of this hypothetical protein whereby it could be implicated in TMA metabolism in *Catenulispora acidiphila*. TMA is a secondary metabolite that can be used by soil bacteria as a primary sole carbon source.

## Electronic supplementary material

Below is the link to the electronic supplementary material.

Supplementary material 1 (TIFF 17162 kb)

## Abbreviations

CaMKII: Ca^2+^/calmodulin-dependent protein kinases II
FTS: Fluorescence thermal shift
KSI: Δ^5^-3-Ketosteroid isomerases
LEH: Limonene-1,2-epoxide hydrolases
MCSG: Midwest Center for Structural Genomics
NTF2: Nuclear transport factor 2
PHZB: Phenazine biosynthesis protein
PDB: Protein data bank
RMSD: Root-mean-square-deviation
SnoaL: SnoaL-like polyketide cyclase
TMA: Trimethylamine
