# Supplementary figures and images for: Structural characterization of a hypothetical protein: a potential agent involved in trimethylamine metabolism in Catenulispora acidiphila
Source: J Struct Funct Genomics. 2014 Feb 22;15(1):33–40. doi: 10.1007/s10969-014-9176-z (PMC3955178; doi:10.1007/s10969-014-9176-z)

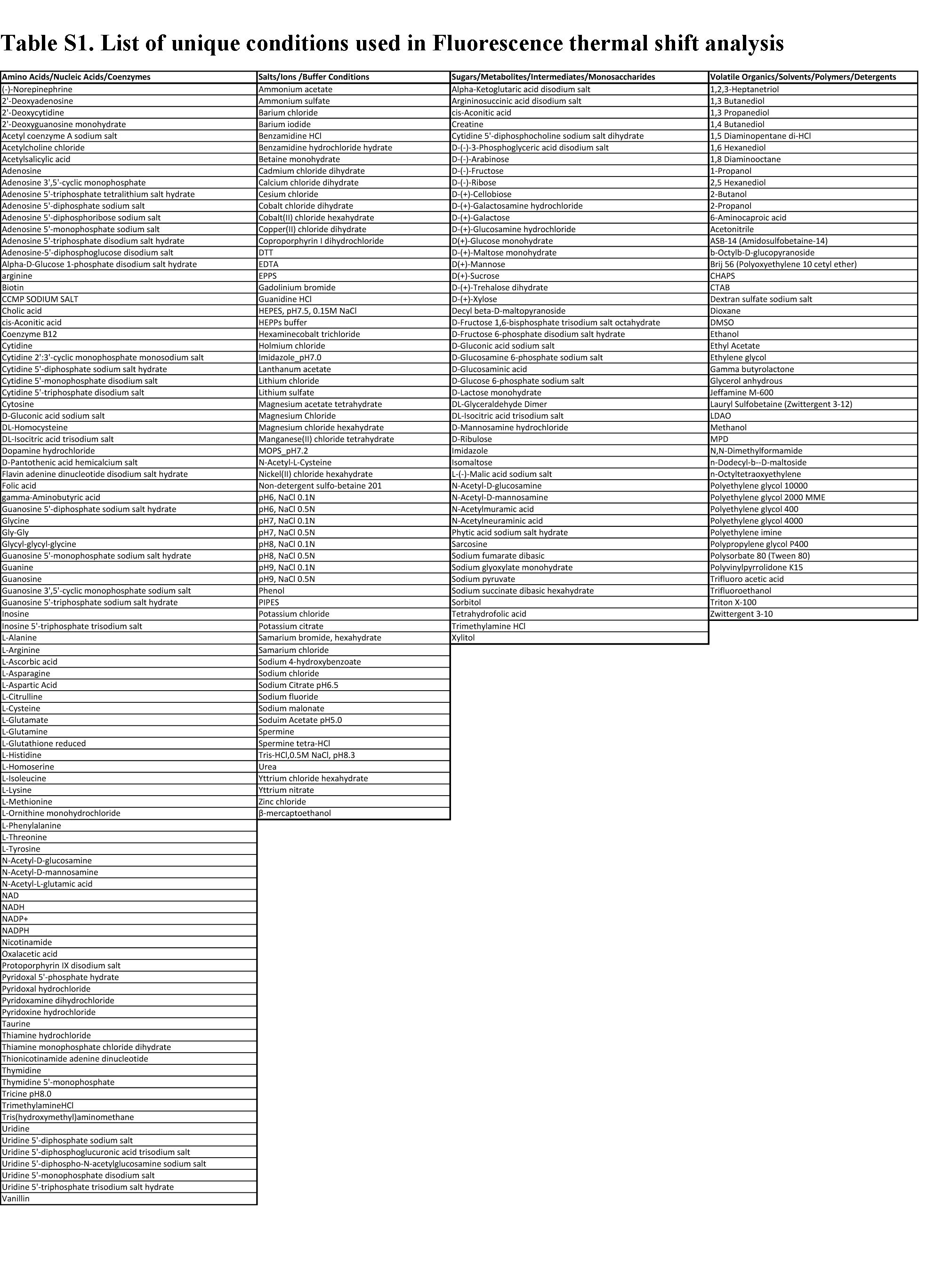

Supplement: Supplementary file 1 — Supplementary material 1 (TIFF 17162 kb) [file 10969_2014_9176_MOESM1_ESM.tif]
